# ATRA sensitized the response of hepatocellular carcinoma to Sorafenib by downregulation of p21-activated kinase 1

**DOI:** 10.1186/s12964-023-01194-1

**Published:** 2023-08-03

**Authors:** Kai Wang, Xun Qiu, Zhensheng Zhang, Hanzhi Xu, Yawen Tan, Renyi Su, Fengqiang Gao, Jianyong Zhuo, Wangyao Li, Zhengxing Lian, Hong He, Xiao Xu

**Affiliations:** 1 Key Laboratory of Integrated Oncology and Intelligent Medicine of Zhejiang Province, Department of Hepatobiliary and Pancreatic Surgery, Affiliated Hangzhou First People’s Hospital, Zhejiang University School of Medicine, Hangzhou, 310006 People’s Republic of China; 2 Westlake Laboratory of Life Sciences and Biomedicine, Hangzhou, 310024 People’s Republic of China; 3 Zhejiang University School of Medicine, Hangzhou, 310058 People’s Republic of China; 4 Department of Surgery, University of Melbourne, Austin Health, Heidelberg, VIC 3084 Australia; 5 Key Laboratory of Integrated Oncology and Intelligent Medicine of Zhejiang Province, Hangzhou, 310006 People’s Republic of China

**Keywords:** ATRA, Chemoresistance, Hepatocellular carcinom, PAK1, Sorafenib

## Abstract

**Background:**

Sorafenib resistance greatly reduces the efficacy of treatments in advanced hepatocellular carcinoma (HCC) patients, but the underlying mechanisms are not thoroughly understood. All-trans retinoic acid (ATRA), an anti-leukaemia agent, has attracted considerable attention due to its role in sensitizing cells to other anticancer treatments. We aimed to investigate the combined effect of ATRA and Sorafenib on HCC and the underlying mechanisms.

**Methods:**

CCK-8, cell sphere formation, trans-well migration, and wound-healing assays were used to analyse the biological behaviours of HCC cells in vitro. Western blotting and qRT-PCR analysis were conducted to measure the expression of p21 activated kinase 1 (PAK1) and phospho-p21 activated kinase 1 (pPAK1). Xenograft models were established to confirm the synergistic effects of ATRA and Sorafenib in vivo. TUNEL assays and immunohistochemistry were utilized to determine apoptosis, proliferation, PAK1 and pPAK1 levels in tumour tissues.

**Results:**

We observed that PAK1 was overexpressed in HCC, and its expression was negatively correlated with the survival of patients. PAK1 promoted the proliferation, self-renewal and epithelial-mesenchymal transition of HCC cells. Correlation analysis indicated that the IC_50_ of Sorafenib was positively correlated with the level of pPAK1 in HCC cell lines. ATRA inhibited the progression of HCC and sensitized HCC response to Sorafenib by downregulation of PAK1, as shown by the calculated coefficient of drug interaction and the data obtained from xenograft models.

**Conclusions:**

Our findings indicated that instead of treatment with Sorafenib alone, the combination of ATRA and Sorafenib provides a more effective treatment for HCC patients.

Video Abstract

**Supplementary Information:**

The online version contains supplementary material available at 10.1186/s12964-023-01194-1.

## Introduction

Hepatocellular carcinoma (HCC) accounts for 90% of primary liver cancer cases, and it is the sixth most common cancer and the third leading cause of cancer-related death worldwide [1]. The 5-year survival of HCC is only 18% [2]. Treatments for HCC include liver transplantation for patients with early stage HCC and surgical resection, radiofrequency ablation, and arterial embolization for patients with advanced stage HCC. In addition, first-line therapy for advanced HCC includes multityrosine kinase inhibitors, such as Sorafenib, and a combination of immunotherapy and antiangiogenic therapy [3].

Sorafenib, a multityrosine kinase inhibitor, is the most widely used first-line therapy in HCC treatment. Sorafenib has prolonged the median survival of patients with advanced HCC from 7.9 months to 10.7 months [4]. However, HCC patients treated with Sorafenib usually acquire drug resistance within a few months, resulting in a poor prognosis [5]. The mechanisms underlying Sorafenib resistance including cancer stemness, tumour microenvironment, epigenetics, transport processes and regulated cell death, are complex [6, 7], and further studies are necessary to understand the molecular mechanisms underlying Sorafenib resistance.

P21-activated kinase 1 (PAK1), a serine/threonine protein kinase, is overexpressed in many cancers including breast, colon, prostate and pancreatic cancers [8]. PAK1 is also implicated in HCC [9]. LncRNA-H19 facilitated the proliferation, migration, and invasion of HCC cells by activating the cdc42/PAK1 pathway [10]. PAK1 accelerated the epithelial-mesenchymal transition (EMT) and proliferation of HCC cell lines through Snail- and β-catenin-dependent pathways [11]. Downregulation of PAK1 suppressed HCC progression in a xenograft mouse model [12, 13]. These previous findings have established the role of PAK1 in HCC growth and metastasis. It is important to further investigate how PAK1 affects therapeutic efficacy in the treatment of HCC.

All-trans retinoic acid (ATRA), an active metabolite of vitamin A, exerts an important effect on embryonic development and various cellular activities [14]. ATRA exerts these effects by binding to nuclear retinoic acid receptors and forming heterodimiers with the retinoid X receptors to promote the transcription of target genes. In addition to genomic regulation, ATRA has also been reported to rapidly modulate multiple signaling pathways (eg., the PKB/AKT, MAPK and PI3K pathways) [14]. ATRA has been found to significantly improve the survival of patients with acute promyelocytic leukaemia [15]. In addition, the combination of ATRA and other anticancer compounds showed synergistic or additive inhibition of the progression of several solid tumours including lung cancer, breast cancer, pancreatic cancer and HCC [16]. C-X-C motif (CXC) chemokines with an NH2-terminal Glu-Leu-Arg motif (eg., CXCL2, CXCL5 and CXCL8) facilitated the infiltration of granulocytic myeloid-derived suppressor cells (G-MDSCs), and were upregulated in non-small cell lung cancer with LKB1 inactivating mutations. ATRA sensitized LKB1-deficient non-small cell lung cancer to anti-PD-1 therapy by inhibiting the proliferation and immunosuppressive function of G-MDSCs [17]. It has also been reported that ATRA synergized with cisplatin in HCC treatment by inducing the differentiation of tumour-initiating cells, a group of cells with stronger self-renewal ability and resistance to conventional radio- and chemo-therapies [18]. We have previously reported that ATRA exerted synergistic effects with gemcitabine to inhibit pancreatic cancer via the downregulation of PAK1 [19]. It would be interesting to determine whether ATRA can enhance the anticancer effect of Sorafenib and the mechanisms involved.

In this current study, the effects of PAK1 on HCC growth and metastasis, and on HCC response to Sorafenib and ATRA treatments, either alone or in combination were determined. We demonstrated that PAK1 expression was negatively correlated with not only the survival of HCC patients but also HCC cell response to Sorafenib treatment (high PAK1 activity reduced the inhibitory effect of Sorafenib on HCC cells), and that the anticancer effect of ATRA alone or in combination with Sorafenib was associated with the downregulation of PAK1. Our results indicate that PAK1 significantly contributed to Sorafenib resistance of HCC and that ATRA sensitized HCC response to Sorafenib in the treatment of HCC via downregulation of PAK1.

## Material and methods

### Cell lines and cell culture

Human HCC cell lines (Hep3B, Huh7 and PLC) were purchased from Shanghai Institute of Cell Biology, Chinese Academy of Sciences. The authenticity of all the cell lines was verified by short-tandem repeat profiling (Invitrogen, CA, USA), and the cell lines were tested for *Mycoplasma* contamination (MycoAlert, Lonza, Basel, Switzerland). All cell lines were cultured at 37 ℃ in an atmosphere containing 5% CO_2_ in MEM or DMEM (Gibco, NY, USA) supplemented with 10% fetal bovine serum (Wisent, Montreal, Canada).

### Reagents

The PAK inhibitor FRAX597 (S7271) and Sorafenib (S7397) were purchased from Selleck (TX, USA). ATRA (R2625) was purchased from Sigma-Aldrich (MO, USA).

### Stable overexpression of PAK1 in Hep3B cells

Lentiviral particles carrying empty vector or PAK1 plasmid DNA were obtained from Genechem (Shanghai, China). Cells were plated in a six-well plate at 30% confluence. Normal media were removed from the wells and replaced with media containing HiTransG A and lentiviral particles. Cells expressing empty vector or PAK1 were selected with 2 μg/mL puromycin (Selleck, TX, USA). and then collected for gene expression assays.

### Real-time quantitative PCR (q-PCR) analysis

Cells were harvested by TRIzol (Invitrogen, California, USA). The cDNA was synthesized using the Hiscript ® II RT SuperMix for q-PCR (+ gDNA wiper) kit (Vazyme, Nanjing, China). Real-time quantitative PCR analysis was performed on a CFX96 Touch real-time PCR system (Bio-Rad, CA, USA) with ChamQ SYBR Color q-PCR Master Mix (Vazyme, Nanjing, China). The amount of target cDNA was analysed through the conversion of the threshold cycle with reference to β-actin. The following primers were used: for human PAK1, forward primer 5'-GGAACCCTAAACCATGGTTCTA-3' and reverse primer 5'-CTCCAGGTAAAATGGATCGGTA-3'; for human β-actin, forward primer 5'-CACCATTGGCAATGAGCGGTTC-3' and reverse primer 5'-AGGTCTTTGCGGATGTCCACGT-3'.

### Western blotting analysis

Total protein lysates were extracted from cells with RIPA buffer (FUDE, Hangzhou, China) supplemented with phosphatase inhibitors and protease inhibitors. Cell lysates were processed by ultrasound and centrifuged at 12,000 rpm for 15 min. The supernatant was transferred to a fresh Eppendorf tube and the total protein concentrations were determined with a BCA protein assay kit (FUDE, Hangzhou, China). The cell lysates were subjected to SDS-PAGE and separated by electrophoresis. The membranes were blocked in 5% non-fat milk and then incubated with the primary antibody overnight at 4 °C followed by incubation with the secondary antibody for 1–2 h at room temperature. The protein bands were visualized by chemiluminescence. The primary antibodies included rabbit anti-β-actin (Abclonal, Wuhan, China), rabbit anti-PAK1 (CST, MA, USA), and rabbit anti-pPAK1 (CST, MA, USA).

### In vitro cytotoxic assay

HCC cells (4 × 10^3^) were seeded in 96-well plates at 100 μl/well. After 24 h, the original medium was replaced by 200 μl medium with or without FRAX597, Sorafenib, or ATRA. CCK-8 assays (MCE, NJ, USA) (MEM or DMEM: CCK-8 = 10:1) were used to assess cell proliferation, and the absorbance was measured at 450 nm. Each experiment was repeated at least three times, and wells without cells were used as blanks. The IC_50_ values were calculated to observe the cytotoxic effects of the drugs. The combined antiproliferative effect of ATRA and Sorafenib combination treatment was evaluated by calculating the coefficient of drug interaction (CDI, CDI < 1, indicates a synergistic effect) [20].

### Colony formation assay

HCC cells were plated in 6-well plates at a density of 800–1,000 cells/well, and the medium was changed every 3 days. After 14 days, the cells were fixed with methanol for 30 min and stained with 0.5% crystal violet for 30 min. The numbers of colonies that had formed were counted by ImageJ.

### Cell sphere formation assay

HCC cells (2 × 10^3^) were placed in ultra-low attachment 24-well plates (Corning, NY, USA) and cultured in DMEM/F12 medium supplemented with B27 (Gibco, NY, USA), 20 ng/ml EGF (Gibco, NY, USA), and 20 ng/ml FGF (PeproTech, NJ, USA). After 8–10 days, cell spheres were collected and centrifuged at 1,000 rpm for 5 min. Cell precipitates were resuspended in 500 ul medium, and 50 ul suspension was added to a 96-well plate for counting.

### Trans-well assay

HCC cells (3 × 10^4^–5 × 10^4^) were suspended in 100 μl medium without fetal bovine serum and loaded onto the upper compartment of a chamber containing a polycarbonate membrane (Corning, NY, USA). Then, 700 μl medium supplemented with 5% fetal bovine serum was added to the lower compartment of the chamber. Forty-eight hours later, the polycarbonate membranes were placed in methanol for 30 min to fix the cells and then stained with 0.5% crystal violet for 30 min. Unmigrated cells in the upper compartment were removed with cotton swabs. The numbers of fixed cells on the membrane were counted.

### Wound-healing assay

HCC cells (2–3 × 10^5^) were seeded into 12-well plates, and after the wells were covered with a monolayer of cells, 3 lines were drawn with 10 μl pipette tips. The lines were photographed under an optical microscope, and the imaged sites were labelled. After incubation in medium without fetal bovine serum for 48 h, images of the labelled sites were captured again. The gaps between the lines were measured by ImageJ.

### Immunohistochemistry

Tumours isolated from mice were fixed with buffered 4% PFA overnight and paraffin‐embedded (Biosharp, Heifei, China). Tumour cell apoptosis and proliferation were measured by TUNEL and immunohistochemical staining for PCNA according to the manufacturer's protocols.

### Animal experiment

All the mice were obtained from the Animal Facility of Zhejiang University. This study (2020SDKS408) was approved by the Ethics Committee of Zhejiang University School of Medicine.

A total of 5 × 10^6^ Huh7 cells suspended in 100 μl PBS or HCC tissues approximately 2 mm in size were subcutaneously injected into the right flanks of five-week-old female NOD-SCID mice. When the tumour volume reached 50–100 mm^3^, the mice were randomly divided into 4 groups (DMSO treatment group, ATRA treatment group, Sorafenib treatment group and combinatorial treatment group, *n* = 3 or 5 in each group). ATRA was intraperitoneally injected daily (10 mg/kg) and Sorafenib was intraperitoneally injected every other day (20 mg/kg) until the tumour volume of the DMSO treatment group was approximately 1000 mm^3^. Each animal was individually monitored throughout the experiment. Tumour size was measured every three days and the volume calculated by the modified ellipsoidal formula: ½ (length × width^2^). The patient-derived xenograft (PDX) models used in this study are from our library [21].

### Statistical analysis

Statistical analysis was performed using GraphPad Prism (version 8.0.1) and R language (version 4.0.3). Comparison of groups was performed with Student’s t-test. Values of *p* < 0.05 were considered significantly different. Pearson's test was used to perform correlation analyses. Kaplan–Meier survival analysis was used to analyse the recurrence-free survival (RFS) and the overall survival (OS) of patients in relation to PAK1 level and a Landmark analysis was used to analyse the effect of time-varying prognostic factors.

## Results

### High levels of PAK1 were correlated with decreased survival of HCC patients

**Fig. 1:**
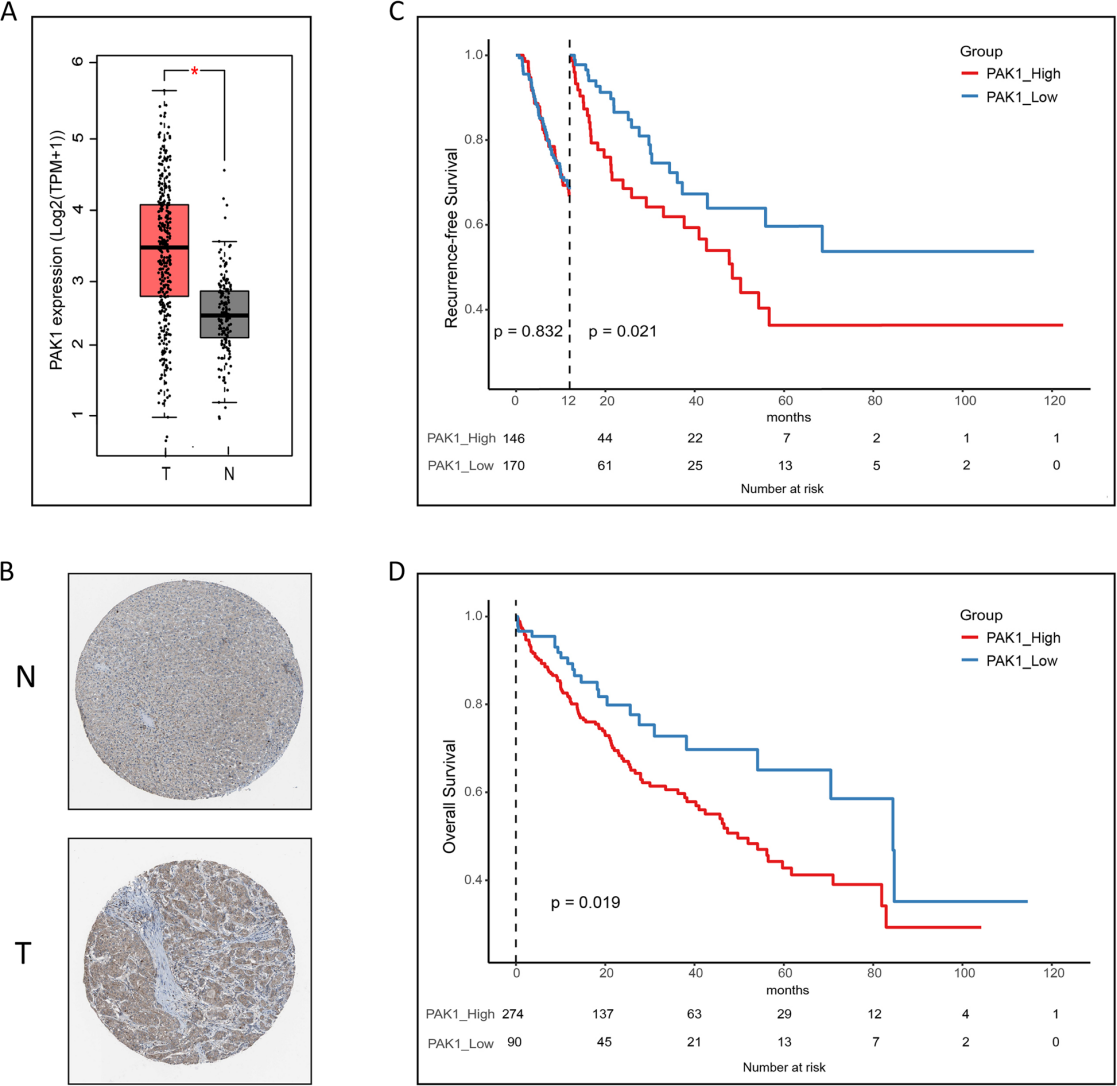
High levels of PAK1 were correlated with decreased survival of HCC patients. **A** Gene expression of PAK1 in HCC tissues (369) and normal tissues (160) from the GEPIA dataset was compared using a t-test. Each dot represented a patient. **B** PAK1 expression in the normal tissue (M-00100) and HCC tissue (M-81703) from the Human Protein Atlas database was determined by IHC. The recurrence-free survival (**C**) and overall survival (**D**) of HCC patients were calculated using Kaplan–Meier and Landmark analysis. N, normal tissue; T, tumour (HCC); TPM, Transcripts Per Kilobase of exon model per Million mapped reads

The results from analysing gene expression in 369 HCC tissues and 160 normal tissues from the GEPIA dataset showed that PAK1 expression was significantly higher in HCC tissues than in normal tissues (Fig. 1A and B). To assess the prognostic relevance of PAK1, the RFS and OS of patients in relation to PAK1 levels were calculated using Kaplan–Meier and Landmark analysis. High levels of PAK1 were correlated with lower RFS and OS in HCC patients (Fig. 1C and D). The results indicated that PAK1 was highly expressed in HCC tissues and that high levels of PAK1 were correlated with decreased survival of HCC patients.

### Activation of PAK1 stimulated proliferation, migration/invasion and anchorage- independent growth of HCC cells

**Fig. 2:**
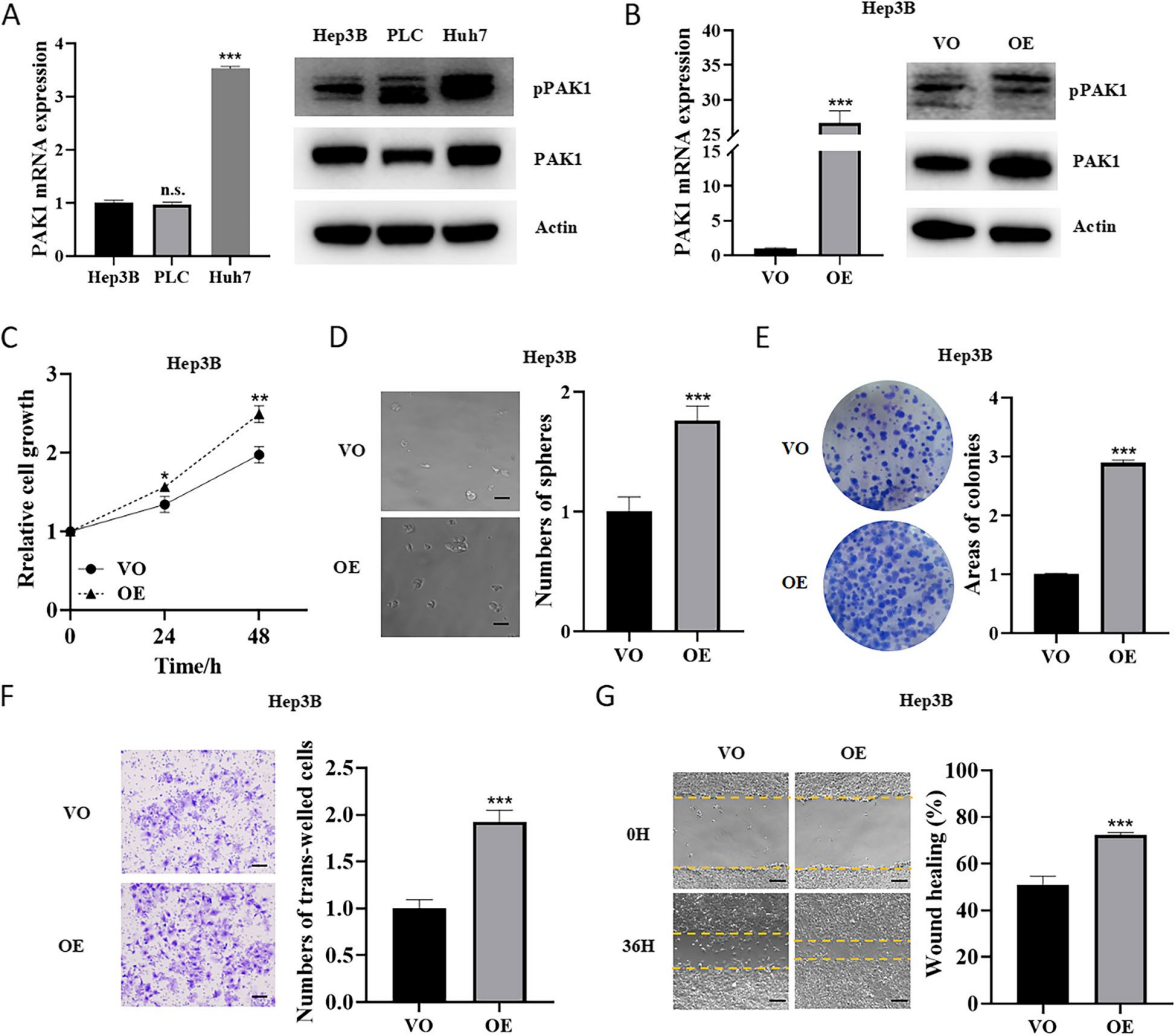
Overexpression of PAK1 promoted proliferation, migration/invasion, and anchorage-independent growth of HCC cells. **A** The mRNA expression of PAK1 and protein levels of PAK1 and pPAK1 in Hep3B, PLC and Huh7 cells were measured by qRT-PCR and Western blotting, respectively. **B** The mRNA expression of PAK1 and protein levels of PAK1 and pPAK1 in a vector-only (VO) clone and a PAK1-overexpressing (OE) clone of Hep3B cells were determined by qRT-PCR and Western blotting. Cell viability (**C**), sphere formation (**D**), colony formation (**E**) and cell invasion (**F**) and migration (**G**) of VO and OE Hep3B cells were measured. n.s., not significant; **p < 0.01, ***p < 0.001, compared with Hep3B or VO. Scale bar, 100 μm. The data are from three independent experiments

To investigate the effect of PAK1 on HCC cells, we first validated the expression of PAK1 and active phosphorylated PAK1 (pPAK1) in different HCC cell lines. The results showed that the level of pPAK1 was highest in Huh7 cells and lowest in Hep3B cells (Fig. 2A). Therefore, Hep3B cells were selected to overexpress PAK1 using lentiviral particles (Fig. 2B). Overexpression of PAK1 stimulated growth (Fig. 2C), and anchorage-independent growth according to sphere formation assay (Fig. 2D) and colony formation assay (Fig. 2E). Furthermore, overexpression of PAK1 enhanced cell invasion shown by trans-well assay (Fig. 2F) and migration shown by wound-healing assay (Fig. 2G). These results indicated that overexpression of PAK1 promoted the growth and invasion of HCC cells.

**Fig. 3:**
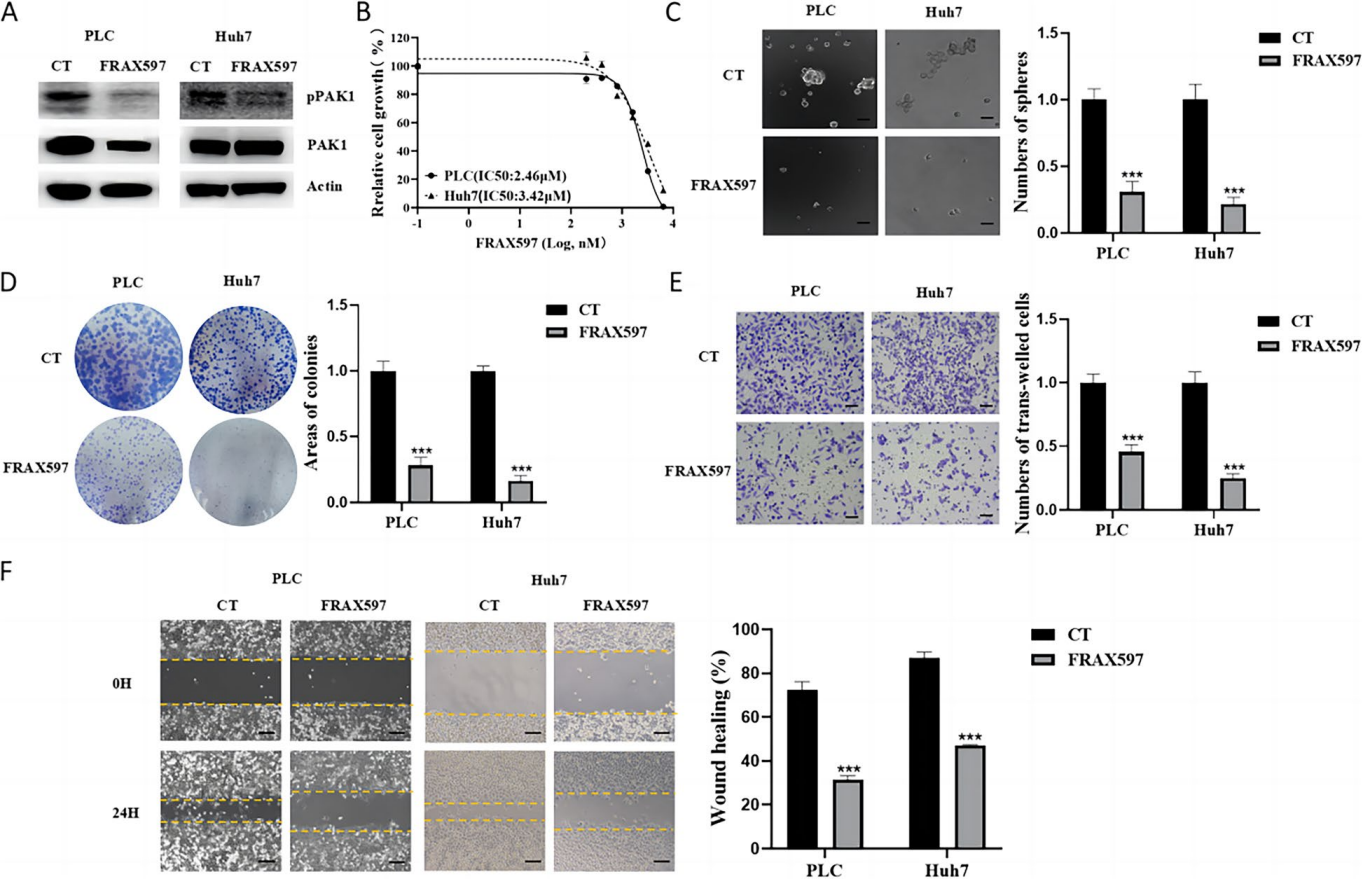
Inhibition of PAK1 by FRAX597 suppressed proliferation, migration/invasion, and anchorage-independent growth of HCC cells. **A** PLC and Huh7 cells were cultured with or without 2 μM FRAX597 for 48 h. The protein expression levels of PAK1 and pPAK1 were measured by Western blotting. Cell viability (**B**), sphere formation (**C**), colony formation (**D**), invasion (**E**) and migration (**F**) of PLC and Huh7 cells treated with or without FRAX597 (2 mM) were determined. CT, control; ***p < 0.001, compared with CT. Scale bar, 100 μm. The data are from three independent experiments

Compared with Hep3B cells, pPAK1 expression was higher in PLC and Huh7 cells. Therefore, PLC and Huh7 cells were treated with PAK1 inhibitor FRAX597 (Fig. 3A). FRAX597 inhibited HCC cell proliferation in a dose-dependent manner by reducing pPAK1 (Fig. 3A and B). As shown in Fig. 3C and D, the number of spheres and colonies formed by HCC cells was significantly decreased in cells treated with 2 μM FRAX597. Furthermore, inhibition of pPAK1 by FRAX597 suppressed cell invasion (Fig. 3E) and migration (Fig. 3F). These results suggested that inhibition of PAK1 by FRAX597 decreased HCC progression by reducing cell growth and migration/invasion.

### PAK1 activity contributed to Sorafenib resistance of HCC cells

**Fig. 4:**
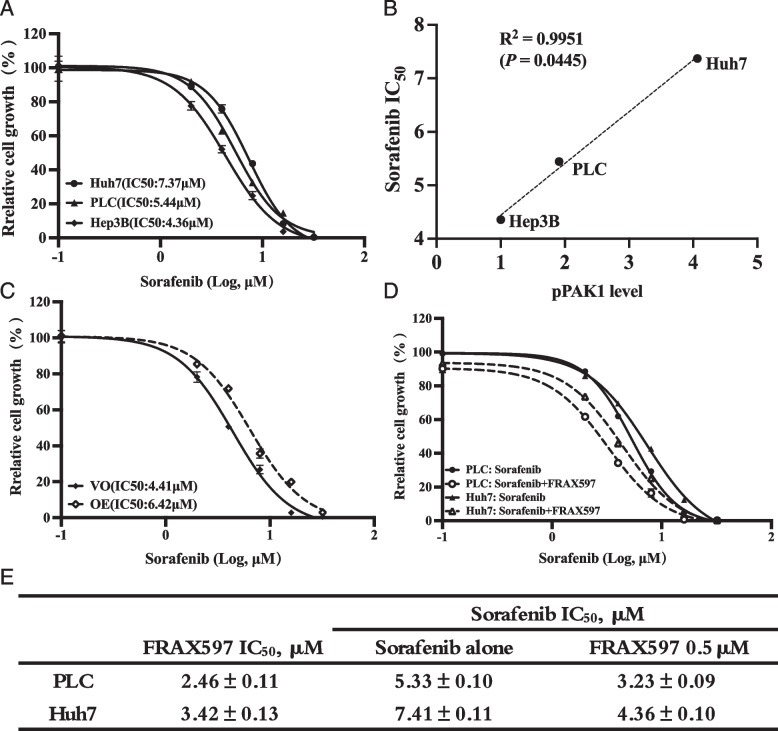
Sensitivity of HCC cells to Sorafenib was negatively correlated with PAK1 expression and activity. **A** PLC, Hep3B and Huh7 cells were treated with Sorafenib (0, 2, 4, 8, 16 and 32 μM) for 48 h, cell growth was determined by CCK-8 assay, and the IC_50_ values were calculated. **B** The correlation between the IC_50_ values of Sorafenib and relative pPAK1 levels in HCC cells was calculated. The growth of a vector-only (VO) clone and a PAK1-overexpression (OE) clone of Hep3B cells was measured in the presence of Sorafenib (**C**) and the IC_50_ values were calculated. The cell growth of PLC and Huh7 cells treated with Sorafenib in the presence or absence of 0.5 μM FRAX597 (**D**) was determined by CCK-8 assay, and the calculated IC_50_ values were listed in (**E)**. The data are from three independent experiments

Given that Sorafenib resistance reduces the efficacy of HCC treatment, we investigated the association between PAK1 and Sorafenib resistance in wild-type cell lines. First, using a cell proliferation assay, we determined the IC_50_ values of Sorafenib in three HCC cell lines with different PAK1 and pPAK1 levels. Our data showed that the IC_50_ value of Huh7 cells was the highest, while that of Hep3B cells was the lowest (Fig. 4A). Correlation analysis indicated that the IC_50_ values of Sorafenib was positively correlated with the relative pPAK1 level (Fig. 4B). Next, the IC_50_ values of Sorafenib in PAK1-overexpression (Fig. 4C) and PAK1-inhibited (Fig. 4D) cells were determined. Consistently, PAK1-overexpressing Hep3B cells were more resistant to Sorafenib and had a higher IC_50_ value than the cells with wild-type PAK1 expression (Fig. 4C), while PLC and Huh7 cells treated with 0.5 μM FRAX597 were more sensitive to Sorafenib and had lower IC_50_ values than their non-treated controls (Fig. 4D). These results suggested that PAK1 activation significantly contributed to the innate resistance of HCC to Sorafenib.

### ATRA suppressed proliferation, migration/invasion, and anchorage-independent growth of HCC cells by decreasing the activation of PAK1

**Fig. 5:**
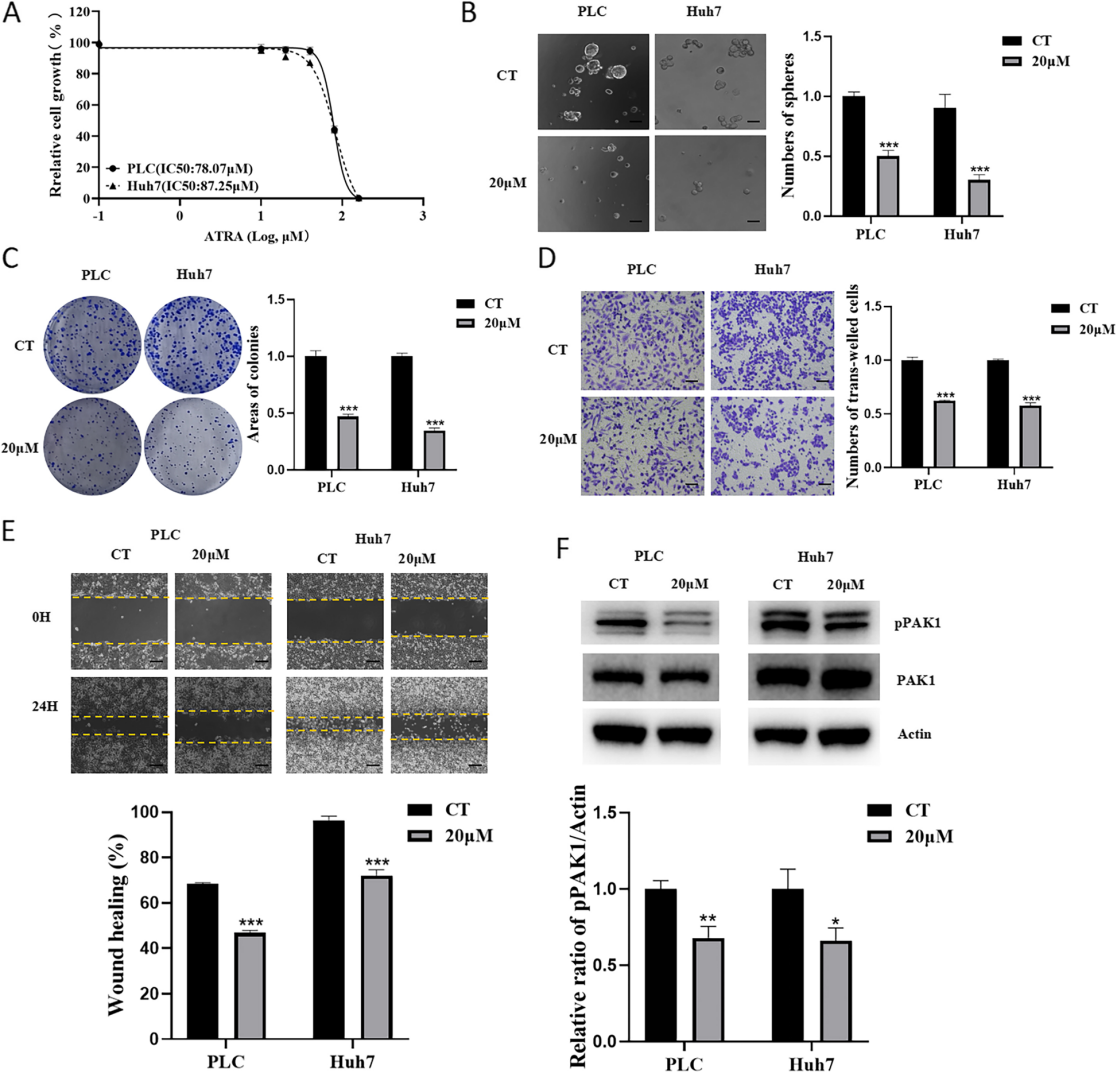
ATRA suppressed proliferation, migration/invasion, and anchorage-independent growth of HCC cells by decreasing PAK1 activation. **A** PLC and Huh7 cells were treated with ATRA (0, 10, 20, 40, 80 and 160 μM) for 48 h, followed by CCK-8 assay to determine proliferation. Cell sphere formation (**B**), colony formation (**C**), invasion (**D**) and migration (**E**) of PLC and Huh7 cells treated with or without 20 μM ATRA were measured. **F** PLC and Huh7 cells were cultured with or without 20 μM ATRA for 48 h. The expression of PAK1 and pPAK1 was analysed. CT, control; ***p < 0.001, compared with CT. Scale bar, 100 μm. The data are from three independent experiments

We further studied whether the anticancer reagent ATRA can inhibit HCC through regulating the activation of PAK1. Both PLC and Huh7 cells were treated with ATRA, and cell proliferation, anchorage-independent growth, invasion and migration were analysed. ATRA repressed cell proliferation in a dose-dependent manner (Fig. 5A). Treatment with 20 μM ATRA markedly inhibited sphere and colony formation of PLC and Huh7 cells (Fig. 5B and C). In addition, HCC cells treated with ATRA displayed reduced trans-well abilities (Fig. 5D) and had a delayed wound closure time (Fig. 5E). Although there was no obvious change in the protein level of PAK1 between the control and ATRA treatment groups, an apparent decrease in pPAK1 was observed in HCC cells treated with 20 μM ATRA for 48 h (Fig. 5F). These results suggested that ATRA inhibited HCC by decreasing PAK1 activation.

### ATRA sensitized the response of HCC to Sorafenib by decreasing PAK1 activity

**Fig. 6:**
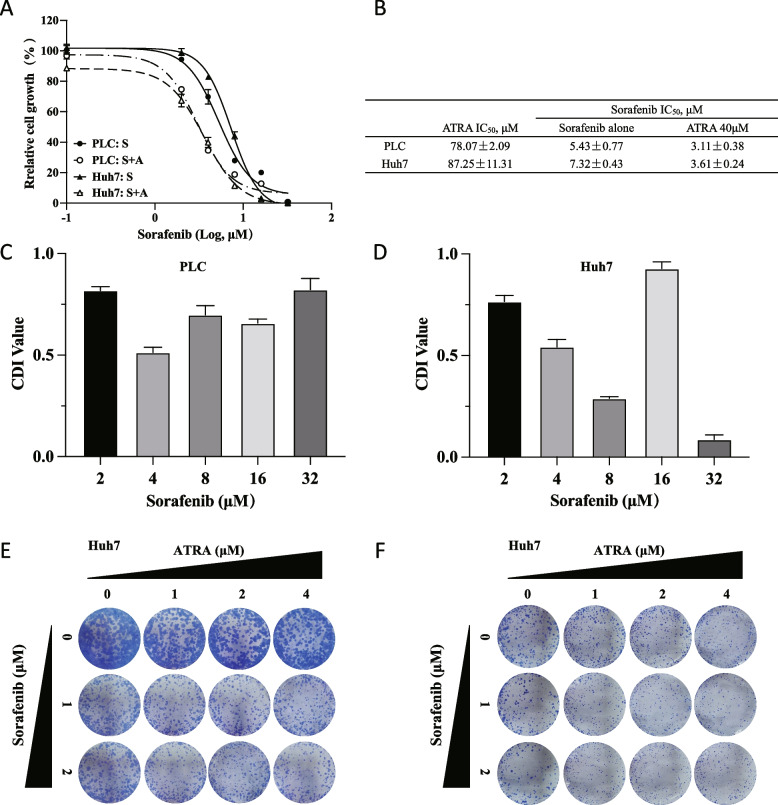
ATRA and Sorafenib synergistically inhibited proliferation and anchorage-independent growth of HCC cells. **A** PLC and Huh7 cells were treated with ATRA (0, 40 μM) and Sorafenib (0, 2, 4, 8, 16 and 32 μM) for 48 h, and cell growth was determined by CCK-8 assay. The IC_50_ values were calculated and listed in (**B**). The coefficient of drug interaction (CDI) calculated using relative viability values for PLC (**C**) and Huh7 (**D**) cells showed the synergistic effect of ATRA and Sorafenib. ATRA and Sorafenib synergistically inhibited the colony formation of PLC (**E**) and Huh7 (**F**) cells. The data are from three independent experiments

To determine the combined effect of ATRA and Sorafenib on HCC, we observed the cell viability and colony formation capacity of HCC cells in response to the combination of ATRA and Sorafenib. As shown in Fig. 6A and B, ATRA markedly reduced the IC_50_ values of Sorafenib in PLC and Huh7 cells. In addition, the calculated CDI values revealed that ATRA and Sorafenib synergistically suppressed cell proliferation (Fig. 6C and D, Tables S1 and S2). Sorafenib combined with ATRA also led to a marked inhibition of colony formation in HCC cells, as evidenced by the reductions in the number and size of colonies (Fig. 6E and F). These results indicated that ATRA can sensitize HCC cells to Sorafenib and promote the anticancer effect of Sorafenib.

**Fig. 7:**
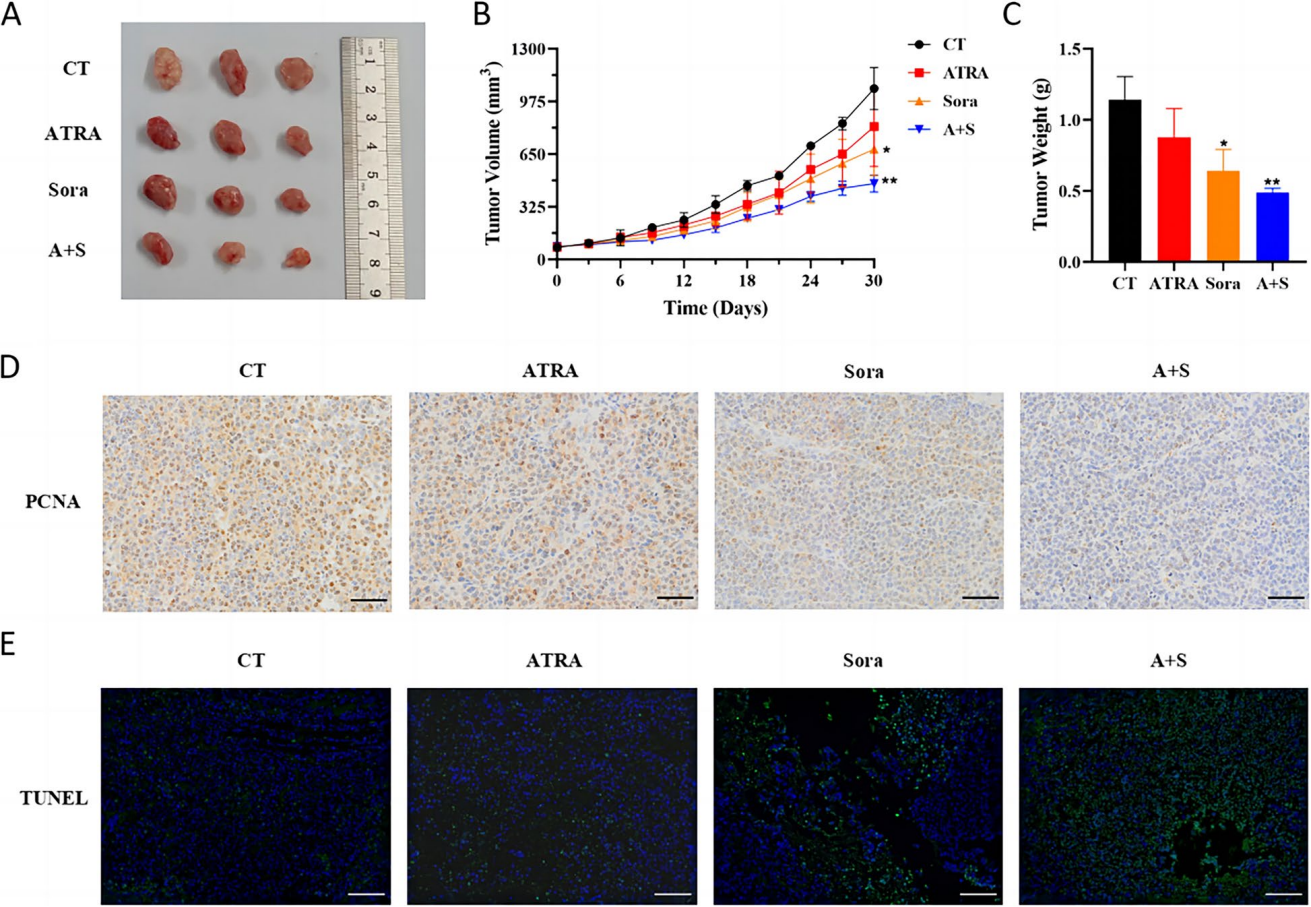
The combination of ATRA and Sorafenib most effectively inhibited cell line-derived xenografted tumor growth. HCC xenografted tumours were treated with ATRA and Sorafenib either alone or in combination as described in the materials and methods. A tumour image (**A**), and the tumour volumes (**B**) and weights (**C**) were presented. Tumour cell proliferation (**D**) was measured by the expression of PCNA in the xenografts as determined by IHC. Tumour cell apoptosis (**E**) was measured by TUNEL assay. CT, control; Sora, Sorafenib; A + S, ATRA combined with Sorafenib; *p < 0.05, **p < 0.01, compared with CT, by t-test. Scale bar, 50 μm

Consistent with the in vitro results, Sorafenib combined with ATRA more significantly inhibited tumour growth compared with the control or either monotherapy in Huh7 cell line-derived xenograft models (Fig. 7A-C). Tumours treated with ATRA and Sorafenib exhibited the lowest proliferative capacity and highest apoptosis (Fig. 7D and E). There was no significant difference in the body weights of the mice among the four groups, indicating that the combination treatment was well tolerated by the mice (Fig. S1).

To determine the role of PAK1 in the effect of the ATRA and Sorafenib combination, we selected two human HCC tissues with remarkably differential PAK1 expression and established PDX models that were treated with ATRA and Sorafenib either alone or in combination (Figs. S2 and S3).

**Fig. 8:**
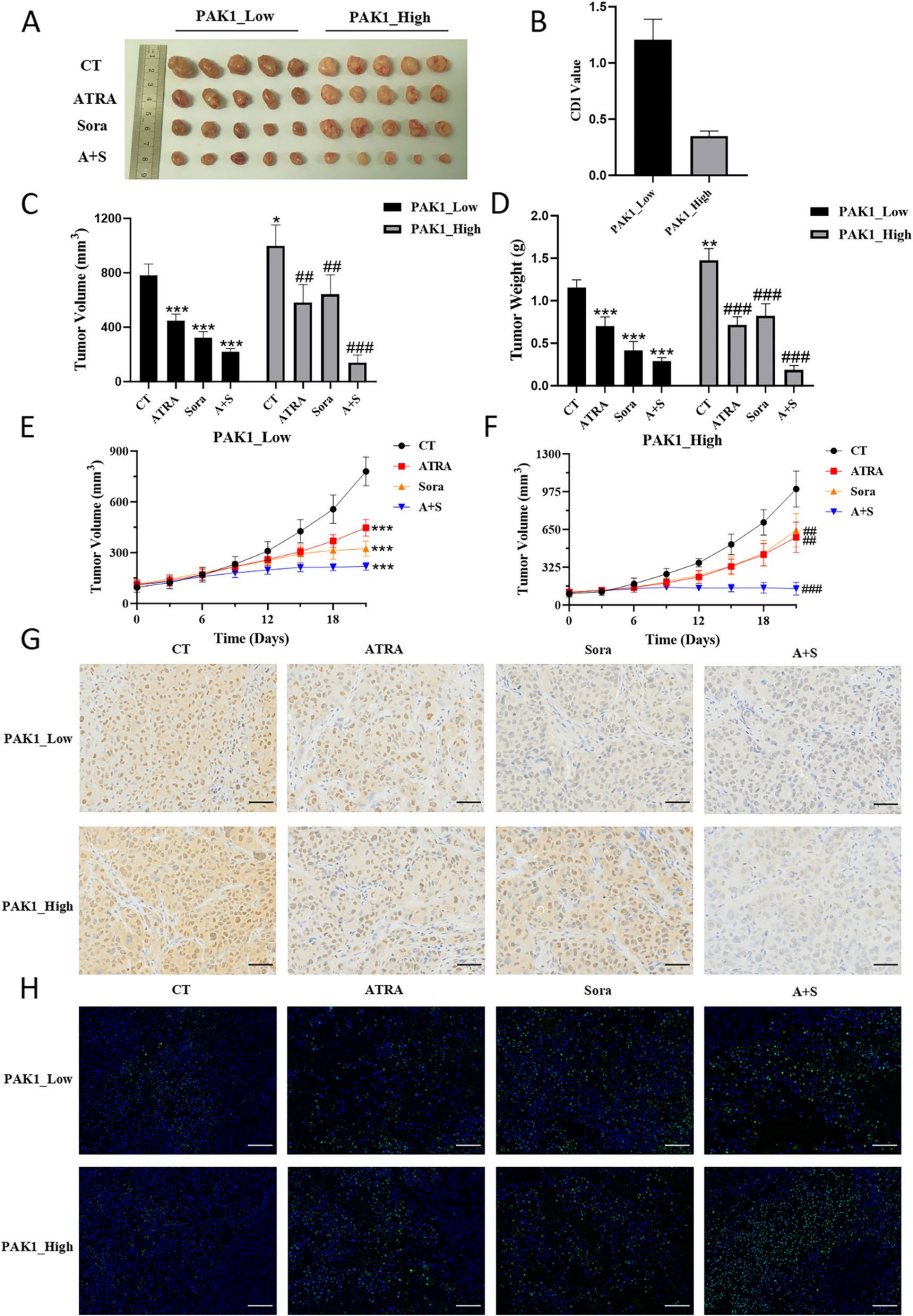
ATRA and Sorafenib synergistically inhibited PDX tumour growth via downregulating PAK1 activity. The tumours were presented in (**A**). The coefficient of drug interaction (CDI) calculated using tumour volume showed the synergistic effect of ATRA and Sorafenib in tumours with high PAK1 levels (**B**). The final tumour volume (**C**), tumour weight (**D**), and tumour growth curves (**E** and **F**) were presented. Tumour cell proliferation (**G**) was measured by the expression of PCNA in the PDXs as determined by IHC. The level of apoptosis in the PDXs (**H**) was measured by TUNEL assay. CT, control; Sora, Sorafenib; A + S, ATRA combined with Sorafenib; *p < 0.05, ***p < 0.001, compared with PAK1_Low CT, ^##^*p* < 0.01, ^###^*p* < 0.001, compared with PAK1_High CT, by t-test. Scale bar, 50 μm

Tumours with high PAK1 levels grew faster and had larger final volumes and weights than those with low PAK1 levels (Fig. 8A-E). Sorafenib monotherapy reduced the final tumour volume by more than 50% in tumours with low PAK1 levels but only by approximately 30% in tumours with high PAK1 levels. However, tumours with high PAK1 levels were more sensitive to the combination treatment than tumours with low PAK1 levels, as evidenced by the reduction in final tumour volume (86% versus 72%) and the calculated CDI values (Fig. 8B and C). These results indicated that high PAK1 activity increased the resistance of HCC to Sorafenib and that ATRA sensitized HCC response to Sorafenib possibly by inhibiting PAK1 activity. Tumour cell proliferation (Fig. 8G) and apoptosis (Fig. 8H) were decreased and increased respectively, by ARTA and Sorafenib either alone or in combination.

## Discussion

PAK1 is upregulated in various cancers, including pancreatic cancer, breast cancer and HCC [10, 22–24]. Dysregulation of PAK1 is associated with drug resistance in several cancers. For instance, PAK1 has been reported to increase pemigatinib resistance in cholangiocarcinoma through β-catenin/Smad4-mediated pathways [25]. The PAK1/β-catenin pathway also plays an essential role in the metastasis of HCC [11]. Inhibition of PAK1 decreased the bone marrow stromal cells-induced resistance to apoptosis in acute myeloid leukaemia [26]. Sorafenib resistance in HCC contributes significantly to the poor prognosis of patients and is mediated by multiple molecular, cellular and tumour microenvironmental mechanisms [6]. It has been reported that a circulating RNA, cirRNA-SORE induced the resistance of HCC to Sorafenib by activating the Wnt/β-catenin signalling pathway [27]. YAP/TAZ, which are transcription factors, drove Sorafenib resistance by repressing Sorafenib-induced ferroptosis [28]. Ferroptosis, an iron-dependent, oxidative form of cell death, plays a key role in the response to Sorafenib in HCC treatment [29]. Moreover, Byun and colleagues recently reported that macropinocytosis, a nutrient-scavenging pathway in certain cancer cells, inhibited Sorafenib-induced ferroptosis, contributing to Sorafenib resistance by activating PI3K-RAC1-PAK1 pathway [30]. Consistently, we reported in this paper that PAK1 activity (pPAK1 level) was positively correlated with the IC_50_ values of Sorafenib in a cell proliferation assay, indicating a role of PAK1 in HCC resistance to Sorafenib. The role of PAK1 in the activation of β-catenin signalling may also contribute to Sorafenib resistance, as reported for pemigatinib resistance in cholangiocarcinoma [25].

We have also shown that ATRA, an approved anticancer reagent, inhibited the activity of PAK1 and suppressed the progression of HCC. ATRA inhibited HCC growth via targeting Pin1, a proline isomerase, and multiple other signalling pathways, including β-catenin, BRaf and AKT [31, 32]. PAK1 functions as a node in the activation of multiple signalling pathways in many cancers [33]. Thus, the inhibition of PAK1 by ATRA may play a key role in the suppression of HCC growth. ATRA inhibited PAK1 in pancreatic ductal adenocarcinoma cells and enhanced the inhibition by gemcitabine of pancreatic ductal adenocarcinoma [19]. In this study, for the first time, we demonstrated that ATRA suppressed the activity of PAK1 and sensitized HCC cells to Sorafenib through the downregulation of PAK1. By inhibiting PAK1, ATRA may block a key node in multiple signalling networks that are required by many cancers and thus suppress cancer growth and enhance the inhibitory effects of other anticancer agents, such as Sorafenib.

Immunotherapy in HCC has shown great progress in the past five years [34, 35]. New clinical trials are exploring combination therapies, including checkpoint inhibitors combined with tyrosine kinase inhibitors such as Sorafenib. However, Sorafenib is still used as one of the first-line treatments for advanced HCC and plays a crucial role in clinical settings [36]. Our finding that PAK1 levels were negatively correlated with the response of HCC cells to Sorafenib indicated an important role of PAK1 in resistance to Sorafenib. Consistently. Our finding that ATRA enhanced the inhibition of HCC by Sorafenib via the downregulation of PAK1 further supported the argument that inhibition of PAK1 attenuates Sorafenib resistance and promotes its therapeutic efficacy in either monotherapy or combination therapy. More importantly, inhibition of PAK1 deceased PD-L1 expression of pancreatic cancer cells and stimulated the antitumour immunity to suppress pancreatic cancer [37]. PAK1 knockout resulted in immune system activation to inhibit intestinal tumorigenesis in a mouse model of intestinal cancer [38]. Inhibition of PAK1 may also stimulate the antitumour immune response in HCC, and thus enhance the efficacy of immunotherapy.

## Conclusions

In summary, we have demonstrated in this paper that HCC patients with high PAK1 expression had shorter survival and that PAK1 was crucial in the tumour growth and metastasis of HCC, contributing to resistance to Sorafenib. ATRA inhibited HCC on its own or enhanced the inhibitory effect of Sorafenib by downregulating PAK1. The combination of PAK1 inhibition with Sorafenib or immunotherapy may approve to be a more effective treatment for HCC.

### Supplementary Information

**Additional file 1: Fig. S1.** Relative to Fig. 7. Body weights of mice in thefour groups.

**Additional file 2: Fig. S2.** Relative to Fig. 8. PAK1 expression in HCC tissuesthat were transplanted into mice.PAK1 mRNA expression in tissues.Proteinlevels of pPAK1 and PAK1 in tissues. ***p<0.001,compared with PAK1_Low.

**Additional file 3: Fig. S3.** Relative to Fig. 8. Body weights of mice in thefour groups.

**Additional file 4: Table S1.** CDI value of ATRA and sorafenib combinations andIC_50_ values calculated from the proliferation assays.

**Additional file 5: Table S2.** CDI value of ATRA and sorafenib combinations andIC_50_ values calculated from the proliferation assays.

## Data Availability

Not applicable.

## Abbreviations

ATRA: All-trans retinoic acid
CDI: Coefficient of drug interaction
CXC: C-X-C motif
EMT: Epithelial-mesenchymal transition
G-MDSCs: Granulocytic myeloid-derived suppressor cells
HCC: Hepatocellular carcinoma
OS: Overall survival
PAK1: P21 activated kinase 1
PDX: Patient-derived xenograft
pPAK1: Phospho-p21 activated kinase 1
RFS: Recurrence-free survival
