# Supplementary figures and images for: ATRA sensitized the response of hepatocellular carcinoma to Sorafenib by downregulation of p21-activated kinase 1
Source: Cell Commun Signal. 2023 Aug 3;21:193. doi: 10.1186/s12964-023-01194-1 (PMC10399044; doi:10.1186/s12964-023-01194-1)

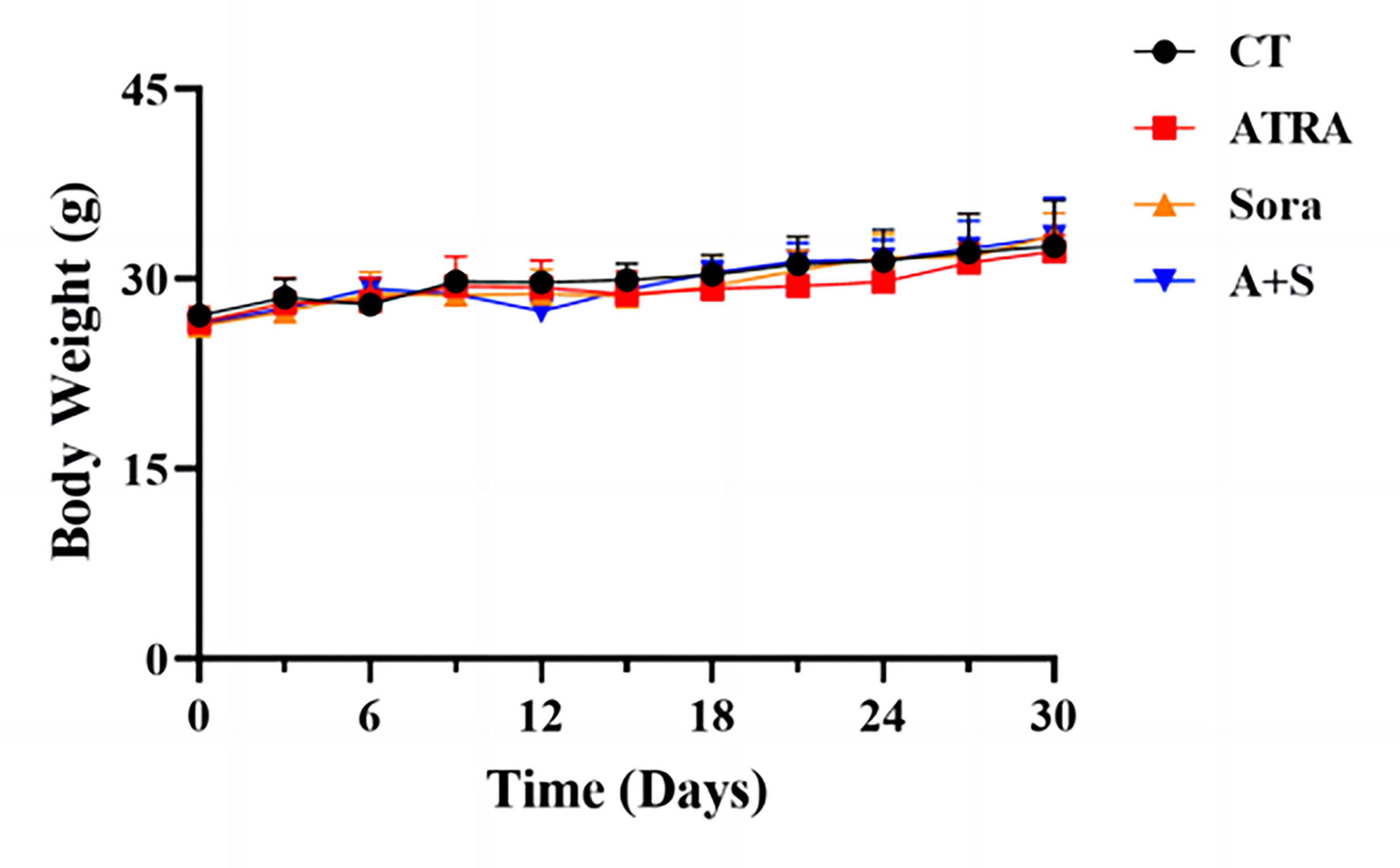

Supplement: Supplementary file 2 — Additional file 1: Fig. S1. Relative to Fig. 7. Body weights of mice in thefour groups. [file 12964_2023_1194_MOESM1_ESM.jpg]

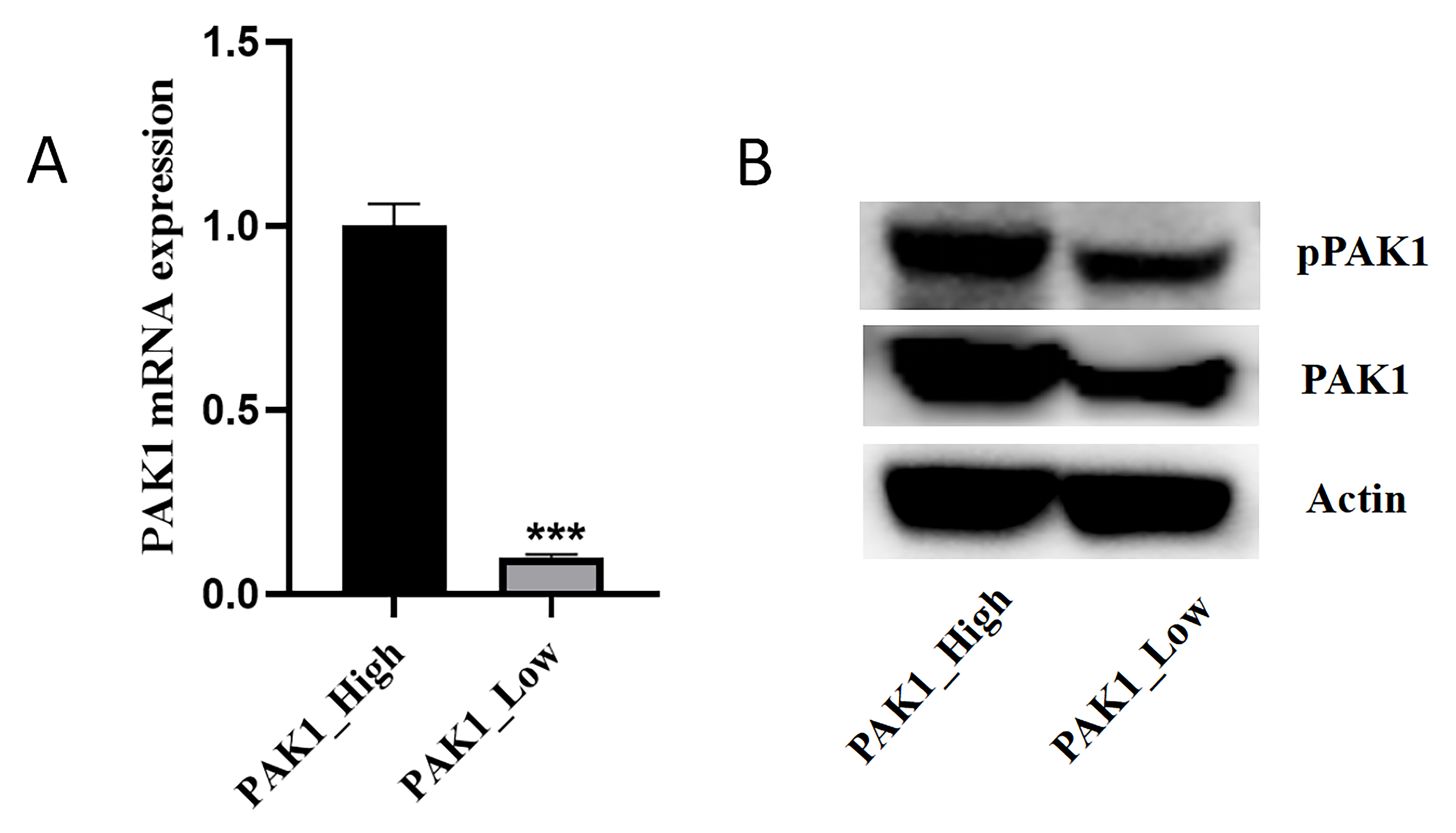

Supplement: Supplementary file 3 — Additional file 2: Fig. S2. Relative to Fig. 8. PAK1 expression in HCC tissuesthat were transplanted into mice.PAK1 mRNA expression in tissues.Proteinlevels of pPAK1 and PAK1 in tissues. ***p<0.001,compared with PAK1_Low. [file 12964_2023_1194_MOESM2_ESM.jpg]

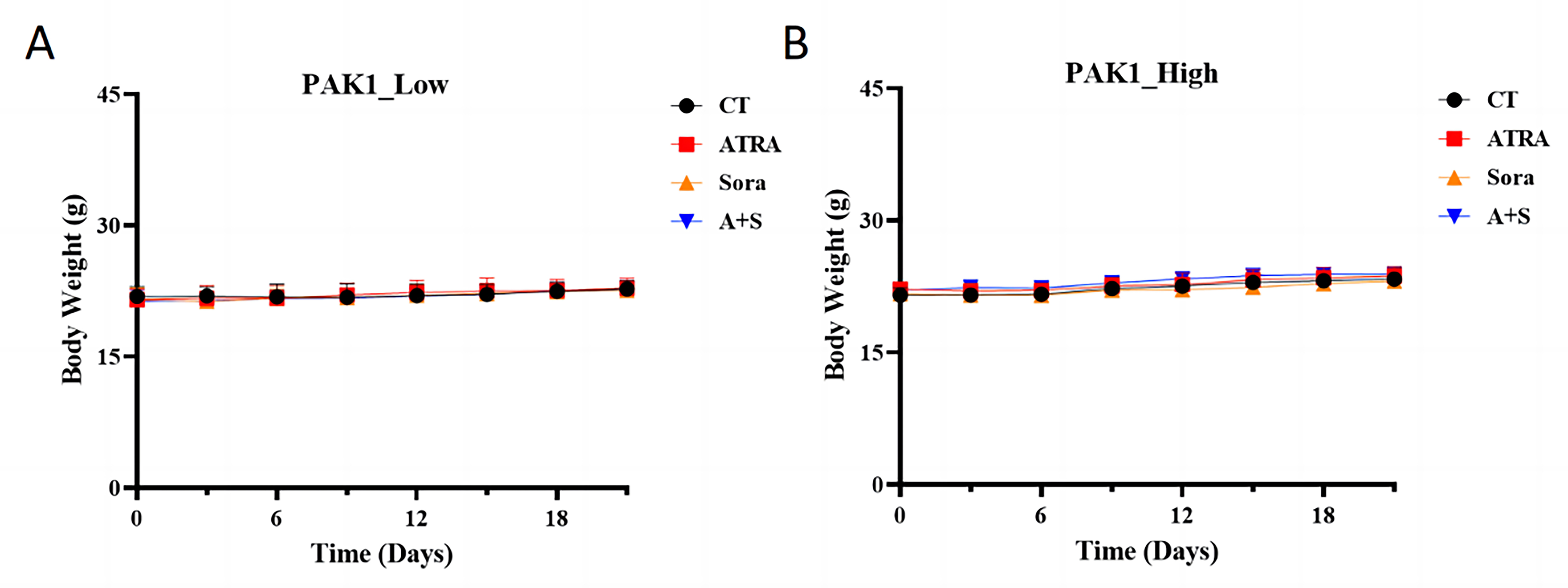

Supplement: Supplementary file 4 — Additional file 3: Fig. S3. Relative to Fig. 8. Body weights of mice in thefour groups. [file 12964_2023_1194_MOESM3_ESM.jpg]
