# Supplementary material for: ATRA sensitized the response of hepatocellular carcinoma to Sorafenib by downregulation of p21-activated kinase 1
Source: Cell Commun Signal. 2023 Aug 3;21:193. doi: 10.1186/s12964-023-01194-1 (PMC10399044; doi:10.1186/s12964-023-01194-1)
Supplement: Supplementary file 5 — Additional file 4: Table S1. CDI value of ATRA and sorafenib combinations andIC50 values calculated from the proliferation assays. [file 12964_2023_1194_MOESM4_ESM.doc]

**Table S1 CDI value of ATRA and sorafenib combinations and IC_50_ values calculated from the proliferation assays.**

| Cell Viability of PLC (%) | | | | |
| --- | --- | --- | --- | --- |
| Concentration (μM) | Sorafenib | ATRA (40μM) | Sorafenib+ATRA (40μM) | CDI |
| Control | 100.59 ± 2.36 | 96.66 ± 1.68 | 100.59 ± 2.36 |  |
| 2 | 94.40 ± 0.09 |  | 74.73 ± 1.40 | 0.82 ± 0.01 |
| 4 | 69.79 ± 3.88 |  | 34.61 ± 0.61 | 0.51 ± 0.02 |
| 8 | 27.89 ± 0.42 |  | 18.85 ± 1.28 | 0.70 ± 0.04 |
| 16 | 20.11 ± 0.57 |  | 12.78 ± 0.39 | 0.66 ± 0.02 |
| 32 | 0.85 ± 0.07 |  | 0.67 ± 0.02 | 0.82 ± 0.05 |
