# Supplementary material for: ATRA sensitized the response of hepatocellular carcinoma to Sorafenib by downregulation of p21-activated kinase 1
Source: Cell Commun Signal. 2023 Aug 3;21:193. doi: 10.1186/s12964-023-01194-1 (PMC10399044; doi:10.1186/s12964-023-01194-1)
Supplement: Supplementary file 6 — Additional file 5: Table S2. CDI value of ATRA and sorafenib combinations andIC50 values calculated from the proliferation assays. [file 12964_2023_1194_MOESM5_ESM.doc]

**Table S2 CDI value of ATRA and sorafenib combinations and IC_50_ values calculated from the proliferation assays.**

| Cell Viability of Huh7 (%) | | | | |
| --- | --- | --- | --- | --- |
| Concentration (μM) | Sorafenib | ATRA (40μM) | Sorafenib+ATRA (40μM) | CDI |
| Control | 102.42 ± 1.56 | 88.59 ± 0.79 | 102.42 ± 1.56 |  |
| 2 | 99.24 ± 1.80 |  | 67.39 ± 3.40 | 0.77 ± 0.02 |
| 4 | 82.81 ± 1.08 |  | 39.85 ± 2.69 | 0.54 ± 0.03 |
| 8 | 44.54 ± 1.90 |  | 11.42 ± 0.45 | 0.29 ± 0.01 |
| 16 | 3.39 ± 0.09 |  | 2.79 ± 0.05 | 0.93 ± 0.03 |
| 32 | 0.13 ± 0.05 |  | 0.01 ± 0.01 | 0.09 ± 0.02 |
